# Moral Competence as a Positive Youth Development Construct: A Conceptual Review

**DOI:** 10.1100/2012/590163

**Published:** 2012-05-02

**Authors:** Hing Keung Ma

**Affiliations:** Department of Education Studies, Hong Kong Baptist University, Hong Kong

## Abstract

Moral competence refers to the affective orientation to perform altruistic behaviors and the ability to judge moral issues logically. A five-stage theory of moral development is proposed. Both western and Chinese perspectives are incorporated in the elaboration of the characteristics of each stage. A brief review of the antecedents of moral competence is presented. The relationship between moral competence and adolescent developmental outcomes is also discussed. Some practical ways to promote moral competence are suggested. School-based programs may be effective in the promotion of moral competence provided it is based on all-round or whole-person development and the length of the program should be sufficiently long.

## 1. Background

We aim to help adolescents develop the competence to act altruistically and to judge fairly. Many contemporary ethical issues have significant implications for education [1]. As more and more people are using Internet every day, we are deeply concerned about the antisocial and disruptive behavior frequently happening on the Internet [2]. A thorough understanding of the concept of moral competence will be useful for the implementation of moral education, whole-person education, and positive youth development education.

## 2. Definition of Moral Competence

Moral competence refers to the affective orientation to perform altruistic behaviors towards others and the ability to judge moral issues logically, consistently, and at an advanced level of development. Promotion of moral competence means fostering the development of justice judgment and altruistic behavior in adolescents. It is the goal of education to help adolescents develop the value of universal justice and universal love.

## 3. Assessment of Moral Competence

Assessment of moral competence depends on the definition and scope of moral competence. For example, an important aspect of moral competence is the altruistic orientation, which can be assessed by Prosocial Orientation Inventory [3], Adolescent Behavior Questionnaire [4–6], and Moral Development Test [7–9]. On the other hand, the stage of moral judgment can be measured by Kohlberg's Moral Judgment Interview [10, 11], Rest's [12, 13] Defining Issues Test, and Ma's [7, 8] Moral Development Test. The assessment of moral competence is a very complicated topic, especially the study of the reliability, validity, and the psychometric properties of the test instruments, and may have to be dealt with in other writings.

## 4. A Chinese Theory of Moral Development

Ma [14–16] has established a theoretical foundation for the psychological study of the affective and cognitive aspects of moral development in Chinese people. The following paragraphs are extracts of Ma's [16] description of the stages of moral development. There are seven stages in the original theory, but only the first five stages are being outlined here because adolescents seldom reach Stage 6 or Stage 7. Details of Stages 6 and 7 are given in Ma's [16] original theory.

Two major parameters underlying the moral development will be discussed in the present model: (i) altruism and human relationships: affective orientation towards others, and (ii) justice: principles for resolving interpersonal conflicts. These two parameters involve to some extent both the affective and cognitive aspects of moral development. However, it is argued that the first parameter is more closely related to some innate human nature and emotion and therefore tends to be more affectively oriented. The second parameter deals with a person's judgment or thinking in moral situations that involve interpersonal conflicts. It has a base on the cognitive developmental approach to morality and thus tends to be more cognitively oriented.

### 4.1. Stage 1: Obedience and Egoism

People at this stage tend to be egocentric, selfish, and obedient to authorities.

#### 4.1.1. Altruism and Human Relationships

**(1) Selfish Orientation**

People at this stage are egocentric and selfish. They tend to seek pleasure and avoid pain, very often at the expense of others. “Unless people look out for themselves, Heaven and Earth will destroy them” (Chinese proverb). It is their intention to get as much as possible out of others but tend to refuse to benefit others or society.

**(2) Parents and Authorities**

They would act altruistically only to gain approval or to avoid punishment by authorities [17, 18]. They would take care of a small group of significant others such as their parents who exert considerable influences on their daily life.

**(3) Survival and Safety Orientation**

People at this stage place emphasis entirely on one's physical survival and safety needs (for a hierarchy of basic needs, see [19]). What is right is to do things that would favor their gratification of survival and safety needs, very often at the expense of others, whether they are in the state of deficiency of basic needs or not. In other words, they would tend to seek out materialistic and lower needs rather than spiritualist and higher needs.

#### 4.1.2. Justice: Principles for Resolving Interpersonal Conflicts

**(1) Obedience to Authorities**

One main reason for a person to obey what the authorities command is to avoid physical punishment.

**(2) Rules as Unchangeable**

For young children, rules are fixed and unchangeable. They would not change a rule because of the intention of the actor nor because of unexpected situational variables. “A rule is therefore not in any way something elaborated, or even judged and interpreted by the mind; it is given as such, ready made and external to the mind” [20, page 106].

**(3) Egocentric Viewpoint**

People at this stage often find difficulty in understanding differences in points of view between themselves and others. In other words, they are not aware of other's reasoning from a third-person perspective. They also confuse the authority's perspective with their own [21].

### 4.2. Stage 2: Instrumental Purpose and Opportunistic Hedonism

The construction of this stage is based on Kohlberg's [17, 22] Stage 2: Individualism, Instrumental Purpose, and Exchange and Loevinger's [23] concept of opportunistic hedonism. People at this stage tend to act with an instrumental purpose to serve their own interests, and they are often Machiavellian in getting what they want. On the other hand, equal exchanges are regarded as fair and acceptable so long as these acts of exchange serve to meet their own needs and interests. 

#### 4.2.1. Altruism and Human Relationships

**(1) Instrumental Purpose**

Acts are usually regarded as instrumental means to serve one's needs and interests. For instance, people at this stage tend to help others who are in desperate situation because they expect others to do the same for them someday. On the other hand, if the situation does not clearly indicate that such help would bring them more benefits than cost to the actor in the long run, then the actor would stick to the rule “mind your own business” or “let things drift if they do not affect one personally,” and he/she would not act to help the victims.

**(2) Significant Others**

People at this stage tend to regard those who are useful to them in the long run and those who would benefit them in one way or another to be their significant others. Parents, spouse, son/daughter, good friends, siblings, and close relatives are examples of significant others. They would act altruistically to these people but not to others. In other words, reciprocal altruism is their guiding principle.

**(3) Opportunistic Hedonism**

People at this stage also believe that “life is a zero-sum game; what one person gains, someone else has to lose” [23, page 17]. It is of course better to gain for oneself and to let others lose. To put it in an extreme form, it means that it is better for others to die and for me to live, if necessary. In other words, they are Machiavellian in maintaining their survival and getting what they want. That is, in order to survive or to get what they want, they would consider using any means, whether the means is legitimate or not. In addition, “work is perceived as onerous. The good life is the easy life with lots of money and nice things” [23, page 17]. The idea is that one should try to get a lot just by paying little or no effort. Generally speaking, people at this stage claim as much rights as they can but tend to bear as little responsibilities as possible. In other words, they act or survive by the principle of opportunistic hedonism.

#### 4.2.2. Justice: Principles for Resolving Interpersonal Conflicts

**(1) Equal Exchange**

People at this stage understand that other people also have similar needs and interests as themselves and therefore they regard equal exchange, fair deal, or reciprocally altruistic behavior as the right act. They also “have a clear sense of fairness as quantitative equality in exchange and distribution between individuals” [17, page 148]. The idea of equal exchange is clearly expressed in the following descriptions. (a) “You shouldn't hurt or interfere with me, and I shouldn't hurt or interfere with you” [17, page 148]. (b) “You scratch my back and I scratch yours.” (c) “You help me today and I will help you tomorrow in return”.

**(2) Concrete Materialistic and Individualistic Perspective**

The contents of the exchange or the deal are often concrete or materialistic things such as money or food, or things which are perceived as good to serve one's own needs or interests such as praise from authorities. The perspective of judgment is individualistic; self-interests precede group or others' interests.

It should be noted that things that are too general or abstract such as basic rights of human beings are seldom considered or valued in the exchange or deal.

**(3) Ignoring Others' Positive Claims and Welfare**

Since people at this stage are holding a concrete individualistic perspective, the positive claims or welfare of others are in general not their concern or responsibility unless such claims and welfare are part of the exchange or deal. In other words, “one has a right to ignore the positive claims or welfare of others as long as one does not directly violate their freedom or injure them” [17, page 215].

### 4.3. Stage 3: Primary Group Affection and Conformity

Primary group refers to family, gang, group of friends or intimates, club, school, party, organization, company and so forth. Generally speaking, members of a primary group share common interests, philosophy, ideology, and in some cases properties. Kohlberg's [17, 22] Stage 3: “Mutual Interpersonal Expectations, Relationships, and Interpersonal Conformity,” and Chinese Cardinal Relationships and family affection are the major bases for elaborating the characteristics of this stage. 

#### 4.3.1. Altruism and Human Relationships

The study of altruism in terms of kin selection and group selection in sociobiology is a good example of primary group altruism. Sociobiology, as defined by Wilson [24], is “the systematic study of the biological basis of all social behavior” [24, page 4]. Its central theoretical problem is “how can altruism, which by definition reduced personal fitness, possibly evolve by natural selection” [4, page 3]? The answer provided by sociobiologists is kinship. As mentioned before, the evolution of altruism involves mainly group selection and kin selection. 

People at this stage are willing to perform altruistic acts towards ingroup members at great sacrifice; however, they are much less willing to do so for outgroup members. 

The Chinese emphasize family integrity, group intimacy, and loyalty. Children are taught to be affective and altruistic to their primary group members [25]. In a cross-cultural study, Ma [7] found that Chinese subjects showed a stronger orientation to perform affective and altruistic acts to first kin, close relatives, best friends than did their English counterparts. The Chinese are also influenced by the concept of the Five Cardinal Relationships of Confucianism which emphasizes a harmonious relationship “between emperor and subject, between father and son, between husband and wife, between brothers, and between friends” [26, page 358]. Generally speaking, the concept of cardinal relationships promotes intimacy and loyalty between oneself and the significant others. In other words, it helps to reinforce primary group altruism. 

People at this stage would consider the gratification of the basic needs of the primary group when they face a dilemma situation. When both they themselves and the primary group are in the state of deficiency of basic needs, they tend to regard the interests of the primary group as important as theirs, or at least as the second important.

#### 4.3.2. Justice: Principles for Resolving Interpersonal Conflicts

**(1) Meeting the Group's Expectation**

People at this stage would live up to “what is expected by people close to you or what people generally expect of people in your role as son, brother, friend, and so forth” [21, page 34]. In other words, the right behaviors are those which can earn approval from the group. In short, it is a “good-boy-nice-girl orientation” [17, page 18].

**(2) The Authority of the Group Leader**

The rules governing the group members' behaviors are often made and administered by the group leaders, sometimes in consultation with the group members. What is right at this stage is then to be loyal to the group, to trust and respect the leaders, and to follow the rules set by the leaders. If conflict occurs, the leaders have final say and people at this stage would suppress or give up their own opinion and stick to the group's rule or the leader's decision. Group order is basically maintained by a style similar to parental control over children.

**(3) Group Rights**

When there is a conflict of interests between the primary group and an individual, people at this stage would think that the rights, whether basic or relative, of the primary group should be protected at the expense of the individual.

**(4) Individual's Responsibility to the Group**

Members of the primary group, in particular the young and junior ones, have the responsibility to contribute to the primary group in order to maintain its survival and prosperity. In other words, the survival of the primary group precedes that of an individual.

### 4.4. Stage 4: Golden Mean Orientation and Social System

The Golden (Happy) Mean is the halfway between two extremes. It refers to a tendency to behave in a way that the majority of people in the society would behave or a tendency to behave in a way that the majority would regard as right or proper. Broadly speaking, consensus, propriety, norms, laws, or social institutions are formed in a way reflecting the general or average opinion, philosophy, rightness, or interests of the majority of people in the society. Thus, consensus, propriety, norms, laws, or social institutions could be regarded as Golden Means. The Golden Mean of Reconciliation, Norm of Filial Piety, and Norm of Social Altruism will be described below. 

#### 4.4.1. Altruism and Human Relationships

**(1) Norm of Filial Piety**

Whether the Chinese like it or not, the social norm prescribes them to uphold strong kinship bondage throughout the whole life span. When they are young, they attach to their parents and grandparents, and, when they grow up into adulthood, they attach to their own children, parents, and sometimes grandparents too. General consensus does not allow the Chinese people to give up their responsibility or filial piety to their aged parents when they grow up, get married, and have their own children. When the norm of filial piety is in conflict with one's interests, one's self-actualization tendency, or other psychological needs, one still has to live up to the norm.

The extension of altruism from primary group to other people in the society has been an important topic in confucianism. For example, in *Liki*, it said, “the teaching of respect to one's elder brothers is a preparation for serving all the elders of the country...” [27, page 131]. The Norm of Social Altruism prescribes people to be altruistic not only to members of their primary group but also to less closely related people in their own society in order to maintain the stability and prosperity of society. It is the social responsibility of every member of society to help each other when in need, not just those closely related with you.

People at this stage would consider the gratification of basic needs of the majority of society in their decision to act in a dilemma situation. They are willing to sacrifice part of their personal interests in order to help those who are deficient of basic needs, in particular deficient of physiological and safety needs.

#### 4.4.2. Justice: Principles for Resolving Interpersonal Conflicts

**(1) Golden Mean of Reconciliation**

Whenever conflict arises, the Chinese tend to resolve conflicts by a soft, tolerating, compromising, and less disturbing attitude. “Reconciliation is precious” is one of the Chinese Golden Means. Such a tolerating and compromising attitude would mean that the Chinese tend to resolve conflicts outside courts or police stations, that is, to resolve conflicts in a less officiated or institutionalized way. “Reconcile big conflicts into small ones, and small ones into none” is the behavior guide of the Chinese at this stage.

**(2) Law-Abiding Perspective**

The behavior of people in a society is usually controlled or constrained by officiated or institutionalized laws. In democratic countries, laws are set up or made by elected representatives of people (e.g., members of Congress or members of Parliament), elaborated or used by court judges and enforced by police. In less democratic countries, laws are made and practiced by a few people in power. In either case, people at this stage would uphold or maintain the laws. What is right is what the law allows and what is wrong is what the law prohibits.

**(3) Social Order and Prosperity**

The general expectation of people in a society is to maintain the stability and prosperity of society. People at this stage would regard anything that contributes to the stability and prosperity of society as right and good. The majority's opinion and interests precede individual's opinions and interests. In contrast to western people who hold an individualistic perspective, Chinese hold a strong collectivistic perspective which gives individuals less freedom to develop their idiosyncratic characteristics and personal opinions. In order words, group orientation and group conformity prevails among Chinese at this stage. People at this stage would think that it is their responsibility to contribute to the stability and prosperity of society.

**(4) Consensus, Norm, and Propriety**

The criteria for differentiating right from wrong are based on general consensus, social norms, propriety, and traditional rules. People at this stage would live up to what is expected by the majority of people in society. Thus, what is right is what the majority regard as right or what the social norms or traditional rules prescribe.

Chinese norms are usually rigid and inflexible, and Chinese are very stubborn in living up to what is expected by the majority, that is, by consensus, norms and propriety. There may be two reasons in accounting for this. (i) The long history of Chinese culture and civilization has left behind lots of virtues, wisdom, and traditions which become a strong basis for formation of social norms and propriety in a Chinese society. These Chinese traditions often become important contents of socialization (e.g., basis for child-rearing and school teaching) for Chinese children. Thus, the Chinese are brought up by rigid and well-established norms which are in bondage to the past. (ii) The face or self-esteem of the Chinese is both strong and delicate; strong in the sense that the face of the Chinese appears to be sacred, invulnerable, and nonnegotiable, and delicate in the sense that it is terribly easy to be hurt. For example, if a person has committed a crime and is caught by the police, then not only would the actor lose his/her face but also his/her parents and close relatives. This kind of act is of course against the public opinions or social norms. It is therefore important to follow closely the social norms in order to keep one's face or maintain one's self-esteem.

### 4.5. Stage 5: Utilitarianism and Basic Rights

The main themes of this stage structure are on the utilitarianism which is concerned with the idea of “seeking the greatest happiness for the greatest number of people” and the basic rights of an individual.

#### 4.5.1. Altruism and Human Relationships

**(1) Utilitarian Altruism**

People act to help by the principle of utilitarianism which aims at the greatest happiness of the greatest number. In other words, if there is a conflict of interest between an individual and the majority, the individual should prepare to sacrifice himself/herself for the majority.

People at this stage feel “a sense of obligation to law because of one's social contract to make and abide by laws for the welfare of all and for the protection of all people's rights” [22, page 175]. In other words, if there is a conflict of interests between the individual and the majority, the individual would act altruistically even at great sacrifices for the majority because of this free agreement and contract.

**(2) Small-I versus Big-I**

The reason for an individual to sacrifice for the majority is based on an affective self-sacrificing altruistic orientation towards the majority. That is, “the small-I should be sacrificed to support the big-I” (Chinese proverb). “Small-I” refers to an individual and “Big-I” refers to the country or the majority of a group. One of the famous ancient Chinese philosophers, Mo Tzu, had proposed a doctrine of universal love, which states that “men should actually love the members of other families and states in the same way that they love the members of their own family and state, for all are equally the creatures and people of God” [28, page 9]. When asked “what good is such a doctrine,” Mo Tzu answered, “it will bring the greatest benefit to the largest number of people” [28, page 10].

People at this stage would consider the gratification of basic needs of the majority as more important than their own gratification of similar basic needs. For example, if both the individual and the majority are suffering from the deficiency of physiological needs, the gratification of the physiological needs of the majority precedes that of the individual.

#### 4.5.2. Justice: Principles for Resolving Interpersonal Conflicts

**(1) Basic Rights and Relative Rights**

Everyone and every society have some basic rights which must be upheld and protected regardless of the opinion of the majority of people. These basic rights are regarded as universal in the sense that every person in every society should have a just or fair claim of these rights. The contents of these basic rights have been elaborated in detail by Kohlberg and his associates. “All citizens have rights to (1) freedom from arbitrary punishment, (2) property, (3) freedom to enter into affiliative or family contracts and relations, (4) fair exercise of authority and political rights to a say in the government, (5) moral respect or dignity, (6) legal justice, (7) freedom to make contractual agreements, (8) access to information, (9) certain civil rights, and (10) a right to life” [29, pages 53-54].

On the other hand, many of the rights, rules, and values are relative to one's group only. “These relative rules should usually be upheld, however, in the interest of impartiality and because they are the social contract” [21, page 35]. Examples of relative rights or values include ritual laws, religious values, traditions, and personal interests.

In comparison to western people, the Chinese at this stage may appear to have a slightly weaker emphasis on political and legal rights. The reason is perhaps that the Chinese believe in moral conscience, ethical principles, and human nature rather than institutionalized law in governing their behavior and maintaining justice and social order. On the other hand, the values, rules, and rights that are relative to Chinese society are plenty and rigid mainly because of its long history of tradition and affective collectivistic perspective.

**(2) Law-Making Perspective**

People at this stage, however, do not blindly maintain the law and social order in all cases. If the law fails to protect a person or a country's basic rights, they would no longer stick to the above social contract and they would urge to make a new law to replace the old one. In some cases, Chinese people at this stage would challenge the authority or the majority with a self-sacrificing altruistic attitude towards those being exploited or the victims in order to maintain justice. They are what we call the “conscientious objectors.” That the Chinese institution and legal system is less rigorous and democratic and that the practice of law depends very much on the subjective and sometimes arbitrary interpretation of the authority and leader would mean that there is no easy way to change a law in a Chinese society.

**(3) Conflict between Majority and Individual**

In general, if there is conflict between (a) the majority's basic rights and an individual's basic rights or (b) the majority's relative rights and an individual's relative rights, then what is right is to sacrifice the individual for the majority or to protect the majority's rights because the majority is composed of a large number of people while the individual is only a single person. The reason is based on an affective self-sacrificing altruistic orientation towards the majority. That is, “the small-I should be sacrificed to support the big-I” (a Chinese proverb). “Small-I” refers to an individual and “big-I” refers to the country or the majority of a group. Everyone in a similar position is supposed to do the same. However, if the conflict is between the majority's relative rights and an individual's basic rights, then the individual must be protected regardless of the majority's opinion. If necessary, a new law has to be made to protect the individual with a socially recognized or institutionalized basis. Similarly, if the conflict is between the majority's basic rights and an individual's relative rights, then the majority must be protected because the basic rights precede the relative rights.

**(4) Social Contract**

The general will, needs, interests, and opinions of the majority of people in a society are either codified into laws or formulated in the form of social norms or proprieties. That everyone should uphold the social norm and abide by the law for the welfare of all is a social contract between an individual and the majority. Social behavior of a person is therefore bound by a social agreement which every rational person would accept. The central theme is that “You are obligated by whatever arrangements are agreed to by due process procedures” [12, page 22].

The present theoretical model has two promising features. First, it attempts to integrate both the affective and cognitive aspects of moral development into one model. The affective aspect is represented by the parameter, *Altruism and Human Relationships,* which has been quite extensively studied by Ma [9, 15, 16]. The cognitive aspect is represented by the parameter, *Justice*, which has its bases of construction on Kohlberg's [17, 22] theory of moral judgment development. Kohlberg's theory is a well-established theory with extensive cross-cultural empirical supports [30, 31]. For future studies, the scope of these two parameters should be widened. It is also possible that more parameters are needed to account for the moral development of the Chinese people. Second, major Chinese thoughts (e.g., confucianism) and western philosophies (e.g., utilitarianism, democracy, and concepts of basic human rights) are incorporated into the model. In addition, the Chinese stages of moral development mentioned above formed the theoretical basis for Ma's empirical studies. Details can be found in his reports [7–9].

## 5. Antecedents of Moral Competence

 Studies showed that parents exert significant influences on the development of moral competence in children and adolescents. “Adolescents agree with parents in the ways they judge moral events and attribute legitimacy to parental authority” [31, page 831]. Research also indicated that the development of prosocial behavior is enhanced by exposure to parental warmth and adult guidance [32]. Ma et al. [5] also found that perceived parental influences by Chinese adolescents in Hong Kong was positively associated with frequency of prosocial behavior and negatively associated with frequency of delinquent behavior. 

Peer interactions are useful and important for the development of morality, empathy, and sympathy in adolescents [32]. In addition, sibling interactions also contribute to the development of perspective taking. In their Hong Kong study of 2,862 Chinese adolescents, Ma et al. [4] found that “antisocial adolescents tended to perceive their best friend as antisocial and exert more negative influences on them, whereas prosocial adolescents tended to perceive their best friends as prosocial and exert more positive influences on them” [4, page 255]. 

 According to Eisenberg et al. [32], research “found that naturally occurring prosocial behaviors in school classrooms (Grades 1 to 12) were relatively rare (only 1.5% to 6.5% of total behaviors)” [32, page 681]. On the other hand, school-based programs deliberately designed to foster the development of prosocial attitudes and behaviors can be effective. Unfortunately, “most programs have involved relatively weak and short interventions that may not be adequate for some groups of children” [32, page 683]. In a study of the longitudinal impact of the project PATHS in Hong Kong, Shek and Yu [33] also found that “participants displayed lower level of substance abuse and delinquent behavior than did the control students” [33, page 546].

## 6. Relationship between Moral Competence and Adolescent Developmental Outcomes

Since the development of moral competence is an important aspect of the psychological development, it is natural that it is positively associated with the other aspects of psychological development such as cognitive development, social development, and emotion development. 

 According to the cognitive developmental approach to morality, cognitive judgment is a component of moral action. In addition, affective and cognitive aspects of moral development are parallel and interrelated. “They represent different perspectives and contexts in defining structural changes” [34, page 349]. The relation between the moral judgment stages and cognitive development stages is also clearly stated in Kohlberg's theory. Kohlberg [35] claims that there is an inner logical order in the sequence of his six stages. That is, “a higher moral stage entails a lower moral stage, at least partly, because it involves a higher logical structure entailing a lower logical structure” [35, page 186]. Kohlberg also argued that the development of moral judgment is dependent on the development of perspective taking [35]. 

 Empathy is another aspect of moral development. Hoffman's [36] development theory postulates four hypothetical levels of empathic response based on the cognitive development of a sense of the other by the child. According to Hoffman [37], empathy is defined as “the involuntary, at times forceful, experiencing of another person's emotional state” [37, page 126].

 Based on Maslow's [19] Hierarchy of Basic Needs, Ma [8] constructed a set of moral orientations indices (e.g., an orientation to love and to be loved by others, an orientation to perform altruistic acts towards others, an orientation to act according to social laws and norms, and an orientation to self-actualize one's potential). He also constructed a set of moral stage indices based on Ma's [15, 16] Chinese theory of moral development. In his empirical study, Ma [8] found that the moral orientation and moral judgment of prosocial adolescents is higher than that of delinquent adolescents. In other words, the development of moral orientation and moral judgment predicts the social behavior pattern of adolescents. 

## 7. Practical Way to Promote Moral Competence in Adolescents

The best and the most practical ways to promote moral competence in adolescents are not to construct a teaching package focusing solely on moral competence. Instead, a comprehensive and all-round positive youth development program is a better choice. The PATHS teaching package [38] which was constructed based on Catalano's [39] 15 positive youth development constructs is a good example. Intervention studies have indicated that the PATHS teaching package has the effect of reducing delinquent behavior [33]. 

Since parents exert tremendous amount of influences on the development of moral competence in adolescents, it is natural that parents should be taught to understand the genuine concept of moral competence, the moral developmental pattern, and the strategies to foster the development of morality in their children. As far as possible, parent education for promoting moral competence in adolescents should be incorporated in any of the educational packages involving moral competence.

A moral environment nurtures moral people, and a corrupt environment tends to tempt people to corruption more easily. In other words, it is important to make the environment as fair as possible and also full of caring and considerate gestures, especially for younger people who are less vulnerable to temptation and less mature in their judgment. Bronfenbrenner [40] argues that a politically pluralistic society favors the development of the higher stage of moral development.

## 8. Conclusive Remarks and Suggestion for Future Research

The concept of moral competence is delineated in detail and a Chinese theory of moral orientation and moral judgment is proposed. While some theoretical and empirical studies have been conducted to support the proposed model, future studies should be conducted to further substantiate the moral stages. In particular, longitudinal study should be carried out to investigate the invariance of the developmental sequence. Moreover, cross-cultural studies are useful to check the cultural universality or cultural specificity of the moral stages. 

It is argued that in order for the school-based program to be successful in helping students to increase their moral and prosocial behaviors and reduce their antisocial behaviors, the program should be based on all-round or whole-person development [41] and the length of the program should be sufficiently long. The two teaching packages constructed in the P.A.T.H.S. project may serve as a good example.
